# Experience, awareness, attitudes and ecological worldviews related to sustainable development among physiotherapy students in Sweden: a cross-sectional survey

**DOI:** 10.1186/s12909-026-09838-9

**Published:** 2026-07-07

**Authors:** Anna Pettersson, Emma Swärdh, Nina Brodin, Annie Palstam

**Affiliations:** 1 Division of Physiotherapy, Department of Neurobiology, Care Sciences and Society, Karolinska Institutet, Huddinge, Sweden; 2 Division of Physiotherapy, Department of Orthopaedics, Danderyd Hospital, Stockholm, Sweden; 3 School of Health and Welfare, Dalarna University, Falun, Sweden; 4 Department of Clinical Neuroscience, Institute of Neuroscience and Physiology, Sahlgrenska Academy, University of Gothenburg, Gothenburg, Sweden

**Keywords:** Consciousness of sustainable development, Education, Health professions education, New Environmental Paradigm, Physiotherapy, Sustainable development, Sustainability Attitudes in Nursing Survey

## Abstract

**Background:**

To achieve a transition toward sustainable development, education is an essential driver of change. Factors like learners' attitudes, cultural beliefs and social communities may influence the learning process. In the field of physiotherapy, there is limited knowledge regarding students' understanding of and attitudes toward sustainable development.

**Aim:**

This study aimed to investigate students’ perceptions related to sustainable development.

**Method:**

All eight undergraduate physiotherapy programs in Sweden were invited to participate. An anonymous online survey was sent out to collect data about (i) Students’ experiences of sustainable development in the curriculum, (ii) Attitudes toward sustainability and climate change in physiotherapy and physiotherapy education, (iii) Ecological worldviews, and (iv) Consciousness of sustainable development.

**Results:**

A total of 116 students responded to the online survey. The results align with previous research on university students showing that students’ perceptions were largely positive. Still, one third of the students stated that the teaching they had received consisted of casual comments or spontaneous discussions and 40% did not support the idea of integrating the subject further.

**Conclusion:**

In general, students demonstrated an eco-centric environmental worldview and awareness of the Sustainable Development Goals. However, their responses also indicated limited exposure to appropriate learning activities related to sustainable development, and their overall perceptions were not uniformly positive. Despite our small sample size, the results may suggest a need to further explore learning activities to support deep learning and scaffolding students in seeing the importance of sustainable development in their education.

## Background

Sustainable development within planetary boundaries, has become an urgent global issue [1]. It recognizes the environment as an essential basis and limit for human society. The United Nations Decade of Education for Sustainable Development [2], led by UNESCO, emphasizes that education is an essential driver of change toward a more sustainable future [2]. The key message of this report is that education should not only increase knowledge but also empower individuals to think critically, act responsibly, and be able to contribute to sustainable societies. Contemporary ways of viewing learning and understanding are based on theories that see learning as an active process shaped by learners' interactions with their social worlds [3]. These theories also posit that learning is fundamentally socially and culturally mediated [4], such that learners' attitudes and cultural beliefs, as well as those of their social communities, will influence the learning process [3]. Considering the ambitions stated in the United Nations Decade of Education for Sustainable Development, this view on learning requires careful consideration, not only in terms of the content of education, but also in terms of student’s perspective, the learning environment and how teaching and learning are organized. In the field of physiotherapy, awareness of the importance of sustainable development within the profession is rising as health care systems face significant challenges due to health threats linked to environmental determinants of health [5, 6]. Therefore, calls have been published to emphasize the role of physiotherapy for sustainable development and sustainable healthcare [5, 7–11]. The fact that healthcare itself contributes to significant emissions that drive climate change [12] underscores the urgent need for healthcare professionals to expand their knowledge, awareness, and ethical engagement in the transition toward sustainable development and healthcare [13–15]. The healthcare professions education, such as physiotherapy, therefore, has a vital part to play in reversing this situation [16].

In Sweden, climate change is on the political agenda that is underpinned by a government action plan. Universities are required by law to promote sustainable development in their activities, ensuring that current and future generations are guaranteed a healthy environment, economic and social welfare, and justice [17]. Although this law has been in force since 2006 progress has been limited [17–19]. This suggests that more effective implementation is still needed.

In a previous study, we found that intended learning outcomes related to sustainable development are largely lacking in physiotherapy undergraduate education in Sweden [20]. Furthermore, teachers fail to integrate content relating to sustainable development and sustainable health care in their teaching [21]. Similar results have been documented in a variety of educational contexts [22–25]. Given that learning is a complex endeavor, aspects other than educational content and pedagogical methods will influence students’ learning, such as the student’s individual characteristics [3, 26]. Students' attitudes may influence their engagement and motivation, both of which are necessary for optimal learning [27]. Studies of students' knowledge, attitudes, and experiences related to sustainable development show that most students find the subject interesting and relevant [28–35]. Yet, some students perceive the educational content on sustainable development as insufficient for their professional roles [28, 33, 35]. Research specifically in the field of physiotherapy education related to sustainable development is however scarce. The limited knowledge regarding students' understanding of and attitudes toward sustainable development, as well as the need to reform education that equips future physiotherapists with the knowledge and ability to provide sustainable healthcare, has provided the rationale for conducting this study. The aim of this study is therefore to investigate students’ attitudes toward sustainable development within physiotherapy and physiotherapy education in Sweden, their ecological worldview and further, to explore students’ awareness of sustainable development.

## Methods

### Study design

This is a cross-sectional, quantitative descriptive study based on a survey conducted with students enrolled in physiotherapy undergraduate programs in Sweden.

### Setting

All physiotherapy programs in Sweden adhere to the Qualification descriptor for Bachelor of Science in Physiotherapy [36].

and comprises 180 higher education credits corresponding to three years of full-time studies spread over 6 semesters. The program is offered by eight higher education institutions in Sweden.

### Participants and data collection

The program directors at all eight undergraduate programs in physiotherapy in Sweden were contacted and invited to participate in the study. Five programs agreed to participate. Upon inclusion, program directors or course leaders were asked to forward the study invitations to students in semester one and six of the programs to participate in an anonymous online survey that took about 10–15 min to complete. An invitation to participate was sent out through e-mail to a total of 837 students during spring and autumn 2023. One reminder was sent out to all students through the program directors or course leaders respectively.

### Ethics approval

The study was reviewed by the Swedish Ethical Review Authority and was determined to be exempt (Dnr 2022–07327-01). The study was conducted in accordance with the Helsinki declaration. Written consent to participate in the study was obtained from all participants before data was collected. They were informed that their participation was voluntary, that they could withdraw at any time and that all identifying information would be anonymized.

### Measures

#### Demographic characteristics

Demographic characteristics collected were age, sex, institution of enrolment, and semester of present studies within the physiotherapy undergraduate program. Four questions related to students’ experiences of sustainable development in the curriculum were specifically designed for this study. These questions covered queries about the extent and nature of learning activities covering sustainable development in which they had participated up to that point in their education (none/occasional comments/spontaneous discussions/some part of a learning activity/whole learning activities). They were also asked whether they were acquainted with Agenda 2030 (yes/no) and if they would be positive to increase the curricular content related to sustainable development (yes/no). Finally, they were asked whether they had engaged in any learning activities related to sustainable development in the previous 12 months (yes/no).

#### Attitudes toward sustainability and climate change in physiotherapy and physiotherapy education

The Sustainability Attitudes in Nursing Survey, (SANS-2) was used to evaluate nurses’ attitudes on sustainability and climate change [37]. It captures participants agreement or disagreement with the significance of climate change and sustainability in nursing, as well as their integration into nursing education, making it potentially relevant for other groups as well. The instrument has been modified and employed within nursing faculty settings [38], among paramedic students [39], and in multiple nursing education contexts across Europe, with evidence supporting good psychometric properties [40]. For the current study, the term “nursing” was replaced with “physiotherapy.” The questionnaire includes five items rated on 7-point Likert scales (1 = strongly disagree to 7 = strongly agree), where higher scores reflect more positive attitudes towards sustainability and climate change (range 1–7). The total SANS-2 score is calculated by averaging the scores of all items (range 1–7). The researchers have obtained permission to use the Swedish version of SANS-2 in this study from the rightsholders [41]. The item interrelatedness (Cronbach’s *α*) of the measure is 0.890 for the total score.

#### Ecological worldviews

The revised New Environmental Paradigm (NEP) scale is a widely recognized tool designed to evaluate individuals’ beliefs and attitudes towards ecological issues and their perspectives on the balance between human activities and the natural world, known as the ecological worldview [42]. The NEP consists of fifteen items that cover five facets: human domination over nature (items 2, 7 and 12); human exceptionalism (items 4, 9 and 14); the balance of nature (items 3, 8 and 13); the risk of an eco-crisis (items 5, 10 and 15), and limits to growth (items 1, 6 and 11). These items are rated on 5-point Likert scales. Agreement with odd-numbered items and disagreement with even-numbered items indicate pro-environmental responses. For even-numbered questions, the scale is: 1 = strongly agree; 2 = mildly agree; 3 = unsure; 4 = mildly disagree; 5 = strongly disagree, with the scale reversed for odd-numbered questions. The NEP reflects either an eco-centred (nature-centred) perspective, where a high NEP score aligns with the view that humans are part of natural systems and emphasizes the intrinsic value of nature, or an anthropocentric (human-centred) perspective, where a low NEP score aligns with the view that humans are separate from or above natural systems and prioritize human needs. The total NEP score is calculated by averaging the scores of all items (range 1–5). The researchers have obtained permission to use the Swedish version of NEP in this study from the rightsholders [29]. The item interrelatedness (Cronbach’s *α*) of the measure was 0.758 for the total score.

#### Consciousness of sustainable development

The Sustainability Consciousness Questionnaire Swedish version (SCQ-S) is a measure created to assess consciousness about sustainable development [43]. It comprises 27 items across three domains: economic, social, and environmental, and evaluates consciousness in these domains through three psychological factors: knowingness (recognition of the importance of sustainability), attitude (attitudes towards sustainability), and behaviour (willingness to act towards a sustainable future). The items are rated on 5-point Likert scales (1 = totally disagree to 5 = totally agree, with a neutral option in the middle), where higher scores indicate greater sustainability consciousness (range 1–5). The total SCQ score for the psychological factors within each domain is averaged to provide a mean total SCQ score (range 1–5). The researchers have obtained permission to use the Swedish version of SCQ in this study from the rightsholders [43]. The item interrelatedness (Cronbach’s *α*) of the measure was 0.843 for the total score.

### Data analysis

All data were analyzed using descriptive statistics of frequencies, means and standard deviations (SD). Missing data were handled by excluding participants with incomplete responses from analyses involving the respective variables, while retaining them in other analyses where data were available. Although the measures were based on Likert-type items, which are ordinal in nature, the scale scores were treated as continuous variables for descriptive purposes, in line with common practice in the literature. The interrelated item of the respective measures was calculated using Cronbach’s *α,* interpreted using a rules-of-thumb threshold for acceptable interrelatedness of α value of at least 0.70 [44]. The assumption of a normal distribution was evaluated using the Shapiro–Wilk test, which is recommended for small to moderate sample sizes. Correlation analyses were performed using Spearman’s rank correlation coefficient, due to minor deviations from normality. The cutoff values for the strength of the correlation used were high (*r* = > ± 0.7), moderate (*r* = ± 0.5–0.7), low (*r* = ± 0.3 −0.5), and negligible (*r* = < 0.3) [45]. All tests were two-tailed with a significance level of *p* < 0.05. The statistical software, SPSS version 28.0.1.1 was used in the analysis.

## Results

A total of 116 students responded to the online survey and were included in the study. Students from all five physiotherapy undergraduate programs were represented in the study sample, whereof 54 (47%) were in the first semester and 62 (53%) were in the sixth semester. Out of the respondents, 83 (72%) were women and 32 (28%) were men. Most respondents (65%) were between 20–30 years old, 26% were between 31–40 years old and 9% were older than 40 years.

Out of the respondents, 32 (29%) reported having experienced at least one whole learning activity or part of a learning activity related to sustainable development in their physiotherapy education, 38 (33%) reported having experienced occasional comments or spontaneous discussions related to sustainable development while 44 respondents (38%) perceived that there was no such content. Out of the respondents, 72 (62%) stated that they had knowledge about Agenda 2030 and the global goals for sustainable development. Sixty-nine respondents (60%) stated that they would like to see further integration of sustainable development within their physiotherapy education program, while 46 (40%) stated that they would not. Thirty-seven (32%) of the respondents reported that they had attended some type of educational activity concerning sustainable development or climate change during the past 12 months.

### Attitudes toward sustainability and climate change in physiotherapy and physiotherapy education

The mean total SANS-2 score for the respondents was 4.9 (SD 1.40). The responses for each item respectively are summarized in Table 1.

**Table 1:** Undergraduate physiotherapy student self-reports on attitudes towards sustainable development and climate change in physiotherapy and physiotherapy education using the Sustainability Attitudes in Nursing Survey, second version (SANS-2), *n* = 115

SANS-2 items	Strongly agree						Strongly disagree	Mean SANS score
	7	6	5	4	3	2	1	
	n (%)	n (%)	n (%)	n (%)	n (%)	n (%)	n (%)	Mean (SD)
1. Climate change is an important issue for physiotherapy	22 (19.1)	12 (10.4)	26 (22.6)	18 (15.7)	21 (18.3)	9 (7.8)	7 (6.1)	4.49 (1.8)
2. Issues about climate change should be included in the physiotherapy curriculum	22 (19.1)	11 (9.6)	22 (19.1)	17 (14.8)	14 (12.2)	19 (16.5)	10 (8.7)	4.24 (2.0)
3. Sustainability is an important issue for physiotherapy	36 (31.3)	23 (20.0)	30 (26.1)	11 (9.6)	9 (7.8)	2 (1.7)	4 (3.5)	5.38 (1.6)
4. Sustainability should be included in the physiotherapy curriculum	31 (27.0)	22 (19.1)	22 (19.1)	18 (15.7)	9 (7.8)	5 (4.3)	8 (7.0)	5.01 (1.8)
5. I apply sustainability principles at home	28 (24.1)	40 (34.8)	28 (24.3)	13 (11.3)	4 (3.5)	2 (1.7)	0	5.60 (1.2)

Missing values: 1 participant had an incorrect questionnaire completion

### Ecological worldviews

**Fig. 1:**
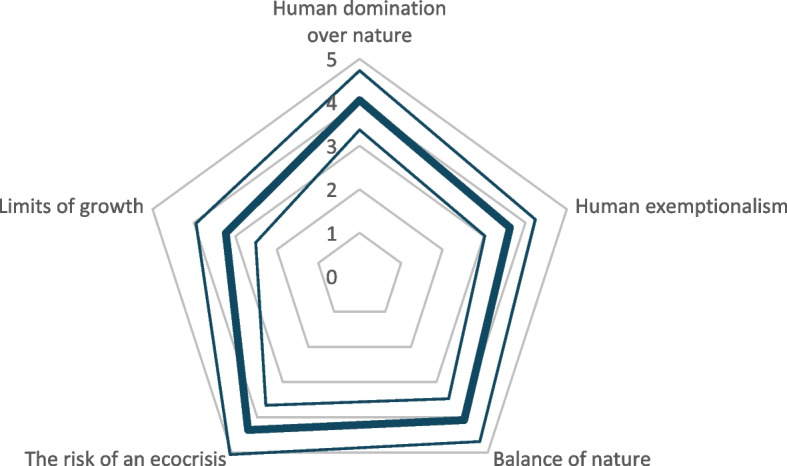
A visual presentation of the five facets of the revised New Environmental Paradigm (NEP) scale. The bold line indicates the mean value while the thinner lines indicate the SD

The total mean score on NEP for the respondents was 3.9 (SD 0.46), indicating an eco-centered ecological worldview. A detailed description of responses for each item respectively is presented in Table 2. The results for each of the five facets of NEP were: Human domination over nature, Mean 4.05 (SD 0.68); Human exemptionalism, Mean 3.63 (SD 0.61); Balance of nature, Mean 4.08 (SD 0.61); The risk of an ecocrisis, Mean 4.36 (SD 0.70), and Limits of growth, Mean 3.23 (SD 0.72). The five facets of NEP are presented in Fig. 1.

**Table 2:** Undergraduate physiotherapy student self-reports on ecological worldviews using the New Ecological Paradigm (NEP) scale, *n* = 113. * For even-numbered questions, the scale is: 1 = strongly agree; 2 = mildly agree; 3 = unsure; 4 = mildly disagree; 5 = strongly disagree, with the scale reversed for odd-numbered questions. The mean NEP score indicates the average degree of pro-environmental responses on a five-point Likert scale (1–5), where higher values consistently reflect more ecocentric attitudes after recoding even and odd questions

NEP items	Strongly agree 1 or 5 *	Mildly agree 2 or 4 *	Unsure 3 *	Mildly disagree 4 or 2 *	Strongly disagree 5 or 1 *	Mean NEP score (SD)
	n (%)	n (%)	n (%)	n (%)	n (%)	
1. We are approaching the limit of the number of people the earth can support	42 (37.2)	40 (35.4)	21 (18.6)	6 (5.3)	4 (3.5)	3.97 (1.0)
2. Humans have the right to modify the natural environment to suit their needs	3 (2.7)	27 (23.9)	12 (10.6)	56 (49.6)	15 (13.3)	3.47 (1.1)
3. When humans interfere with nature it often produces disastrous consequences	46 (40.7)	55 (48.7)	5 (4.4)	5 (4.4)	2 (1.8)	4.22 (0.9)
4. Human ingenuity will ensure that we do NOT make the earth unlivable	0	17 (15.0)	46 (40.7)	32 (28.3)	18 (15.9)	3.45 (0.9)
5. Humans are severely abusing the environment	73 (64.6)	36 (31.9)	3 (2.7)	1 (0.9)	0	4.60 (0.6)
6. The earth has plenty of natural resources if we just learn how to develop them	38 (33.6)	39 (34.5)	26 (23.0)	8 (7.1)	2 (1.8)	2.09 (1.0)
7. Plants and animals have as much right as humans to exist	75 (66.4)	27 (23.9)	5 (4.4)	4 (35)	2 (1.8)	4.50 (0.9)
8. The balance of nature is strong enough to cope with the impacts of modern industrial nations	1 (0.9)	4 (3.5)	21 (18.6)	43 (38.1)	44 (38.9)	4.11 (0.9)
9. Despite our special abilities humans are still subject to the laws of nature	45 (39.8)	37 (32.7)	25 (22.1)	3 (2.7)	3 (2.7)	4.04 (1.0)
10. The so-called “ecological crisis” facing humankind has been greatly exaggerated	3 (2.7)	10 (8.8)	18 (15.9)	24 (21.2)	58 (51.3)	4.10 (1.1)
11. The earth is like a spaceship with very limited room and resources	26 (23.0)	47 (41.6)	20 (17.7)	11 (9.7)	9 (8.0)	3.62 (1.2)
12. Humans were meant to rule over the rest of nature	2 (1.8)	12 (10.6)	6 (5.3)	36 (31.9)	57 (50.4)	4.19 (1.1)
13. The balance of nature is very delicate and easily upset	32 (28.3)	51 (45.1)	17 (15.0)	13 (11.5)	0	3.90 (0.9)
14. Humans will eventually learn enough about how nature works to be able to control it	6 (5.3)	10 (8.8)	45 (39.8)	38 (33.6)	14 (12.4)	3.39 (1.0)
15. If things continue on their present course, we will soon experience a major ecological catastrophe	65 (57.5)	33 (29.2)	11 (9.7)	2 (1.8)	2 (1.8)	4.39 (0.9)

Missing values: 3 participants had incorrect questionnaire completions

### Consciousness of sustainable development

**Fig. 2:**
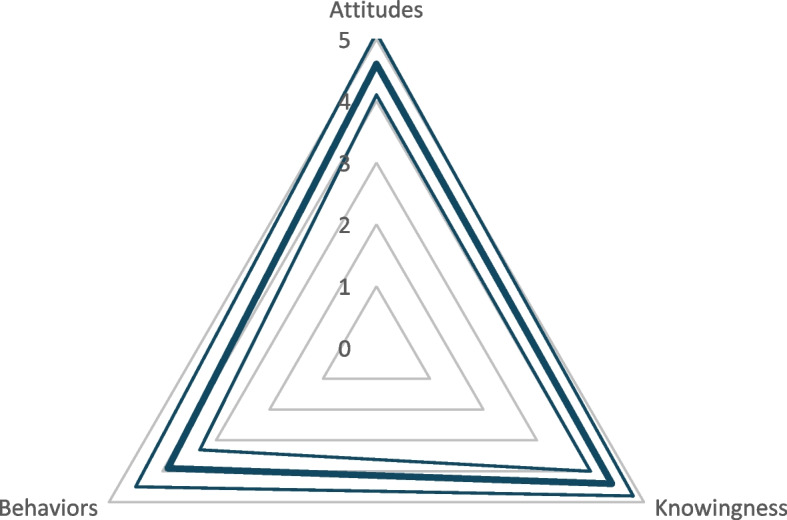
A visual presentation of The Sustainability Consciousness Questionnaire Swedish version (SCQ-S). Knowingness, attitudes and behaviours related to economic, social and environmental consciousness of sustainability. The bold line indicates the mean value. The thinner lines indicate the SD

The total mean score for the undergraduate students’ consciousness of sustainable development SCQ was 4.3 (SD 04), *n* = 115. The highest mean score was for the subscale *Attitudes* with a mean of 4.6 (SD 0.5), followed by the subscale *Knowingness* with a mean of 4.4 (SD 5.4) and the subscale with the lowest score was *Behaviors* with a mean of 3.9 (SD 0.6) Fig. 2.

### Correlations

There was a negligible correlation (*r* = 0.212, *p* = 0.025) between having a strong ecological worldview and having more positive attitudes towards sustainability and climate change in physiotherapy education. There was a low correlation (*r* = 0.415, *p* < 0.001) between having a strong ecological worldview and having high consciousness of sustainable development. There was a low correlation (*r* = 0.499, *p* < 0.001) between having high consciousness of sustainable development and having positive attitudes towards sustainability and climate change in physiotherapy education.

## Discussion

This study explored physiotherapy students’ experience of, and attitudes toward sustainable development in education, their ecological worldview, and their awareness of sustainable development. Perceptions and attitudes regarding sustainable development have been examined among university students and in health professions education [25, 29, 30, 32, 34, 35] but is not widely researched specifically in the field of physiotherapy. Some of the results in the present study are particularly noteworthy. First, the results align with previous research on university students, which show largely positive attitudes toward and perceived relevance of sustainable development issues [25, 29, 30, 32, 34, 35]. However, a closer examination of the result paints a fragmented picture which can pose challenges both from a learning perspective and in terms of educational leadership. While students generally demonstrated an eco-centric environmental worldview and awareness of the Sustainable Development Goals, their responses also indicated limited exposure to appropriate learning activities, and their overall perceptions were not uniformly positive. For instance, one third of the students stated that the teaching they had received consisted of casual comments or spontaneous discussions, and 40% did not support the idea of integrating the subject further. Given education’s role in fostering critical and responsible engagement for sustainability, such learning activities are inadequate. Niemi et al. [33] reported that nursing and medical students experienced weak links between their education and the SDGs and sought greater clinical relevance. The low correlations observed suggest that, while a stronger ecological worldview or greater consciousness of sustainable development is associated with perceiving these issues as important, the relationship remains weak. This may indicate that students’ personal ecological values are only weakly reflected in their views on the relevance of sustainability content within physiotherapy education. In other words, endorsing sustainability at an individual level does not necessarily mean that students see it as a priority within their professional training. This potential disconnect could reflect uncertainties about the professional relevance of sustainability. It may also indicate a lack of confidence in how such topics relate to physiotherapy practice and education. This finding is consistent with previous research among physiotherapy educators, where similar challenges in linking sustainability to professional practice have been described [21]. Although the overall Sustainability Consciousness Questionnaire score was high, the Behavior subscale scored lower than both Attitudes and Knowledge. A similar pattern was found in a study by Abasiyanik et al. [28] among physiotherapy students and professionals. While students’ attitudes are widely recognized as influential for learning processes [3], the relationship between positive attitudes and actual behavior appears to be more complex and non-linear. As previous research suggests, individuals often struggle to translate sustainability-related values and intentions into consistent, sustainable actions [46, 47].

These results align with earlier findings of previous research showing limited integration of sustainable development in Swedish physiotherapy curricula and teachers’ reported lack of confidence and pedagogical strategies in this area [20, 21]. This suggests a need for review of pedagogical strategies to support constructive alignment and deep learning. A recent scoping review by Carrion et al [23] noted a growing body of literature presenting a variety of educational initiatives related to sustainable development but also highlights an absence of standardized conceptual frameworks, curricular content and pedagogical approaches. The planetary health education framework could provide such standardized framework for the education of health professionals [48]. Still, it needs to be broken down and specified further to be relevant for physiotherapists specifically. To profoundly change attitudes and, consequently, behavior, transformative learning has been proposed as an educational strategy [49]. Most likely, successful change requires a long-term and inclusive approach including as new ways of thinking about teaching and learning as well as pedagogical leadership [49–52]. Student-centered learning environments and student influence, together with professional development for teachers and clear institutional support, have been suggested as key elements [50, 52].

While interpreting the result, several aspects should be considered. The collected data reflects variation in gender, year of study, and educational institution, which strengthens the study’s ability to identify patterns in students’ perceptions and helps to better understand students’ attitudes toward sustainable development within physical therapy education. The study provides a transparent and detailed description of its context, methodology, and analysis, which facilitates assessment of the findings’ transferability. However, transferring the results to other contexts, settings, or groups should be approached with caution. The response rate was low, and the findings are based on cross-sectional data of self-reported perceptions. Also, although the data collection was anonymous, self-reports should be interpreted with some caution. First, voluntary participation carries a risk of skewing the sample towards participants already positive from the outset which may give the false impression that students are more positive than they actually are. Nevertheless, the result in this study reflects both positive and negative attitudes. In addition, questions about the environment could be perceived as a sensitive topic and linked to personal values, morals, or political ideology. Respondents may therefore have answered in a way that either downplays undesirable views or adjusts their response to what they believe is more socially acceptable [53, 54]. Furthermore, having an overall positive attitude toward sustainable development issues does not necessarily imply a thorough understanding of the concept [32, 34, 35, 47]. The response rate in this study was below 14%, which represents a significant limitation and should be carefully considered when interpreting the findings. A low response rate increases the risk of non-response bias, as individuals who chose not to participate may systematically differ in their experiences, awareness, and attitudes compared to respondents. Consequently, the results may not fully capture the diversity of perspectives within the target population. The limited engagement may reflect survey fatigue or a lack of interest in the topic, which in itself could be indicative of how sustainable development is currently perceived within the educational context. The measures used in the study were adapted from a nursing context and had not been specifically validated for use within physiotherapy prior to their application in this study. However, the only modification to the questionnaire was the professional label and given that nurses and physiotherapists function within similar clinical contexts and share responsibility for promoting sustainable healthcare, this adaptation is unlikely to have substantially altered the underlying construct being measured. Previous studies indicate that SANS demonstrates generally stable psychometric properties across different cultural and educational contexts. This said, it does not entirely rule out the possibility of profession-specific interpretations. Another aspect to be mindful of when interpreting the result is the wider sociocultural context where data was collected. Sweden counts as a high-income country where access to education is very good and highly valued in society, [55]. Sustainable development and climate change are on the political agenda and are frequently discussed and debated in public discourse. There is legislation that requires all authorities, including education at all levels, to conduct their activities in line with sustainable development. Reasonably, these conditions may have influenced students’ awareness of the issue and are reflected in the respondents' answers.

## Conclusion

In general, students demonstrated an eco-centric environmental worldview and awareness of the Sustainable Development Goals. However, their responses also indicated limited exposure to appropriate learning activities related to sustainable development, and their overall perceptions were not uniformly positive. Despite our small sample size, the results may suggest a need to further explore learning activities to support deep learning and scaffolding students in seeing the importance of sustainable development in their education.

## Data Availability

The datasets used and/or analysed during the current study are available from the corresponding author on reasonable request.
